# Multifidus lesions: A possible pathological component in patients with low back pain after posterior lumbar surgery

**DOI:** 10.1097/MD.0000000000037239

**Published:** 2024-03-01

**Authors:** Dan Pan, Ke Liu, Meiyuan Huang, Tiansheng Sun, Zhicheng Zhang

**Affiliations:** a Department of Spinal Surgery, Zhuzhou Central Hospital, Zhuzhou, Hunan, China; b Department of Pharmacy, Zhuzhou Central Hospital, Zhuzhou, Hunan, China; c Department of Pathology, Zhuzhou Central Hospital, Zhuzhou, Hunan, China; d The Second School of Clinical Medicine, Southern Medical University, Guangzhou, Guangdong, China.

**Keywords:** Low back pain, Multifidus, posterior lumbar surgery, TGF-β1

## Abstract

There are few histological studies on multifidus after lumbar surgery, and it is not clear whether multifidus changes affect the clinical outcome after lumbar surgery. The aim of this study was to investigate the relationship between multifidus changes and clinical outcomes after lumbar surgery. Patients underwent internal fixation removal after lumbar posterior surgery were enrolled. Patients were divided into a low back pain (LBP) group (*n* = 15) and a non-low back pain (non-LBP) group (*n* = 10).The Oswestry disability index (ODI) and visual analog scale (VAS) were completed. 18 patients with lumbar fracture surgery were included as the control group. Multifidus morphological changes were observed by hematoxylin and eosin and Masson staining. The expression of TGF-β1 was observed by immunohistochemistry, immunofluorescence and Western blot. The cross-sectional area (CSA) of the multifidus in the non-LBP group and the control group were greater than those in the LBP group. TGF-β1 expression and gray value ratio in the non-LBP group and the control group were lower than those in the LBP group. The multifidus CSA and TGF-β1 expression in multifidus were strongly correlated with ODI and VAS. Patients with LBP after posterior lumbar surgery suffered from atrophy and fibrosis lesions in the multifidus, and the degree of multifidus lesions was closely related to dysfunction and pain, which might be one of the causes of LBP after posterior lumbar surgery.

## 1. Introduction

Lumbar disc herniation, lumbar spinal stenosis, lumbar spondylolisthesis, and degenerative lumbar scoliosis are common spinal disorders, and surgical treatment usually should be considered when conservative treatment is ineffective. Posterior lumbar decompression, fixation and fusion surgeries, including posterior lumbar interbody fusion (PLIF) and transforaminal lumbar interbody fusion (TLIF), are the most commonly used surgical strategies for the treatment of lumbar degenerative diseases. Most patients receive relief of low back and leg pain after surgery. However, some patients may complain of severe low back pain (LBP) rather than leg pain or numbness after lumbar surgery. As a common complication after lumbar surgery, there are many causes of postoperative LBP, including internal fixation failure, inadequate surgical decompression and improper selection of patients, as well as the presence of pain or dysfunction prior to surgery, negative personality, anxiety, depression, and negative postoperative rehabilitation process.^[1]^ In some patients, postoperative imaging suggest adequate decompression, precise fixation, good fusion, and overall and local balance, and the cause of postoperative LBP fail to be identified.^[2]^

Lumbar spine surgery causes histological changes in the paraspinal muscles. Kawaguchi et al^[3]^ performed paraspinal muscle biopsy in patients with poor prognosis after posterior lumbar surgery and found that paraspinal muscles were dysfunctional due to local denervation, atrophy, and loss of support, increasing biomechanical strain, and leading to failed back surgery syndrome.^[1]^ The most affected structure after posterior lumbar surgery is the multifidus, as it is closest to the posterior midline of the spine, directly apposed to the lamina, and is a central component of the spinal stabilization system.^[4]^ Fibrotic lesions severely impair skeletal muscle function by reducing motion and contractility. Transforming growth factor-beta (TGF-β) is a multifunctional cytokine, including 3 isoforms of TGF-β1 to 3. Activation of the TGF-β signaling pathway triggers collagen accumulation and is important for wound repair, but overexpression of TGF-β results in enhanced extracellular matrix and progressive fibrosis, thus winning the title of “the master regulator of fibrosis.”^[5]^ TGF-β1 is one of the most widely studied isoforms, and is elevated in plasma, serum, and muscle tissue of patients with different skeletal myopathies. Overexpression of TGF-β1 contributes to fibrotic tissue formation and dysregulated muscle regeneration.

Previous studies on the multifidus after posterior lumbar surgery have mainly focused on histological changes, with rare study on fibrosis. As an ideal indicator for the evaluation of muscle fibrosis, TGF-β1 expression in multifidus has not been studied, and no study reports that whether multifidus lesion increases the risk of poor prognosis after lumbar surgery. In this study, we selected multifidus condition after posterior lumbar surgery as the observation object to investigate the effect of multifidus changes on the prognosis of posterior lumbar surgery.

## 2. Materials and methods

### 2.1. General data

Patients who visited our hospital from September 2018 to September 2020 and received the second posterior lumbar surgery for removal of lumbar internal fixation were selected. Inclusion criteria: Patients with severe low back and leg pain before the first surgery, which affected daily work and life, and failed to respond to conservative treatment for more than 3 months; patients were diagnosed with lumbar disc herniation or lumbar spinal stenosis, and the choice of the first surgical approach was a single-level PLIF or TLIF procedure from L3 to L5; Imaging examination before the second internal fixation removal surgery without abnormal signs such as insufficient surgical decompression, level error, unstable fixation, poor fusion, lumbar instability, adjacent degeneration, infection, and severe osteoporosis. The patients were divided into the LBP group and non-low back pain (non-LBP) group according to whether they had chronic LBP before the second operation. Criteria for defining chronic LBP^[6]^: Oswestry disability index (ODI) ≥ 20 and visual analogue scale (VAS) ≥ 4; length of LBP ≥ 3 months or total time of LBP ≥ 3 months in the past 6 months; pain is described as persistent pain located in the lumbosacral region but not radiating to the legs, which can be aggravated by standing, sitting, walking, bending over, and relieved by bed rest. The sex, age, body mass index (BMI), chronic LBP before the first operation or not, ODI and VAS before the first operation, length of medical history before the first operation, treatment of level for the first operation, ODI and VAS before the second operation, and interval between the 2 operations were recorded in the 2 groups.

About 15 patients were included in the LBP group, including 7 males and 8 females. The age ranged from 38 to 66 years, with an average age of 52.20 ± 9.70 years. BMI 16~29 kg/m^2^, average 22.27 ± 4.23 kg/m^2^. There were 11 patients with chronic LBP and 4 patients without chronic LBP before the first operation. ODI before the first operation ranged from 12% to 49%, with an average of 29.67% ± 10.43%. VAS was 2 to 8 points before the first operation, with an average of 5.47 ± 1.80 points. The first preoperative history was 15 to 48 months, with an average of 28.30 ± 10.24 months. The surgical segment was located at L3/4 in 5 cases and L4/5 in 10 cases. The ODI before the second operation ranged from 20% to 39%, with an average of 29.80% ± 6.66%. VAS 4 to 8 points before the second operation, with an average of 5.87 ± 1.41 points. The interval between the 2 operations was 16 to 45 months, with an average of 27.87 ± 9.40 months.

The non-LBP group included 10 patients, 6 males and 4 females. The age ranged from 39 to 65 years, with an average age of 52.60 ± 8.66 years. BMI 15~30 kg/m^2^, average 21.27 ± 4.69 kg/m^2^. There were 7 patients with chronic LBP and 3 patients without chronic LBP before the first operation. The ODI before the first operation ranged from 10% to 42% (mean 31.00 ± 10.88%). VAS was 2-8 points before the first operation, with an average of 5.81 ± 1.87 points. The first preoperative history ranged from 12 to 60 months, with an average of 31.80 ± 15.60 months. The surgical segment was located at L3/4 in 3 cases and L4/5 in 7 cases. ODI before the second surgery ranged from 6% to 16%, with an average of 10.00% ± 3.37%. VAS 1.00 (0.75 ~ 1.25) before the second surgery. The interval between the 2 operations was 18 to 40 months (mean 27.40 ± 6.75 months).

Patients who came to the hospital due to traumatic lumbar fracture at the same period were selected as the control group. Inclusion criteria: Fracture located in L2-L4 vertebral body, completed the posterior midline approach of the lumbar spine fracture reduction and internal fixation surgery in 1 week after injury; in 1 level below the fracture, MRI showed no injury of the posterior musculo-ligamentous complex; patients without previous history of low back or leg pain or related spinal surgery. Indicators such as gender, age, and BMI were recorded for patients in the control group. About 18 patients were included, including 5 males and 13 females. The age ranged from 34 to 65 years, with an average age of 46.11 ± 8.32 years. BMI 17~29 kg/m^2^, average 22.40 ± 3.95 kg/m^2^. Fracture segments were located at L2 in 12 cases, L3 in 4 cases, and L4 in 2 cases.

This study has been approved by the Ethics Committee of Zhuzhou Central Hospital of Hunan Province, China. All patients informed the study risks and benefits and signed the informed consent form.

### 2.2. Sample collection

In the LBP group and the non-LBP group, the superficial multifidus specimens were taken from the original surgical site, and in the control group, the superficial multifidus specimens were taken from the 1 level below fracture site, with a bundle of muscle tissue about 0.5 cm × 0.5 cm × 2 cm in size, and samples were frozen in liquid nitrogen for future use.

### 2.3. Treatment of samples and study methods

Routine hematoxylin and eosin (HE) and Masson’s trichrome staining was used to determine histological changes in muscle specimens. Under higher power magnification, 10 discontinuous visual fields of muscle specimens for each section were selected randomly by the Leica image acquisition system (Leica Microsystems, Bannockburn, IL). About 10 muscle fibers were selected from each field of view for the HE stained section, and the cross-sectional area (CSA) of each muscle fiber was calculated by Image J software version 1.8.0 (National Institutes of Health, Bethesda, MD), following which, the mean CSA was automatically calculated. The ratio of myofibers to collagen fibers of each field of view from the Masson trichrome staining section was calculated by Image J (NIH, Bethesda, MD), and then the mean ratio was automatically calculated.

Immunohistochemistry and immunofluorescence staining were performed on paraffin embedded sections as reported previously and the muscle specimens were sectioned at a thickness of 3 μm. The sections were blocked at room temperature using saline containing 0.1% BSA and 10% goat serum. Rabbit anti-TGF-β1 (Bioss, Beijing, China) and horseradish peroxidase (HRP)-labeled goat anti-rabbit IgG (H + L) (Zsbio, Beijing, China) were used for immunohistochemistry. Rabbit anti-TGF-β1 (Bioss, Beijing, China) and goat anti-rabbit IgG Cy3 conjugates (Cwbio, Beijing, China) were used for immunofluorescence analysis. About 10 discontinuous visual fields were also randomly selected under the Olympus microscope and fluorescence microscope (Olympus Co., Tokyo, Japan) for each section respectively. The integrated optical density (IOD) of TGF-β1 expression in each visual field was determined using Image J, and then the mean IOD was automatically calculated.

Western blot was used to examine the protein expression levels of TGF-β1. Tissues were homogenized in ice-cold modified RIPA buffer. The supernatants were collected after centrifugation, separated on a 12% sodium dodecyl sulfate/polyacrylamide gel, and electrotransferred onto a polyvinylidene fluoride membrane. The membranes were incubated in blocking solution for 1 hour at room temperature and then overnight at 4°C with the following primary antibodies: TGF-β1 (Bioss, Beijing, China), and anti-GAPDH (Zsbio, Beijing, China). Membranes were then incubated with appropriate horseradish peroxidase–conjugated secondary antibody for 2 hours at room temperature (Zsbio, Beijing, China). Reactive bands were visualized by using a UVP AutoChemi System (UVP, Cambridge, UK). Semiquantitative measurement of bands was performed by densitometric analysis using Image J. Protein expression levels were normalized to GAPDH.

### 2.4. Statistical analysis

Statistical analysis was performed by SPSS 17.0 software. The Shapiro–Wilk test was used to judge whether the data fit a normal distribution. Statistical differences were analyzed using the chi-square test, *t* test, or analysis of variance (conforming to normal distribution), or Wilcoxon signed rank test (not conforming to normal distribution). Correlations were performed using Pearson correlation coefficient for bivariate normally distributed data and Spearman correlation coefficient for nonconforming bivariate normally distributed data. *R* < 0.3 was considered weak correlation, *R* = 0.3 to 0.5 moderate correlation, *R* = 0.5 to 0.7 strong correlation, and *R* > 0.7 significant correlation. The multifidus histology and TGF-β1 expression were compared between the groups, and correlation analysis was performed for the relationship between multifidus morphology, TGF-β1 protein expression and chronic LBP and dysfunction in the LBP group. The test level was α = 0.05, and *P* < .05 was considered statistically significant.

## 3. Results

### 3.1. Comparison of general data

The age and BMI of the 3 groups conformed to the normal distribution. The ODI and VAS before the first operation, the length of medical history before the first operation, the ODI before the second operation, and the interval between the 2 operations in the LBP group and non-LBP group conformed to the normal distribution. The VAS before the second operation in the LBP group conformed to the normal distribution, and the VAS before the second operation in the non-LBP group did not conform to the normal distribution. There was no significant difference in gender, age, and BMI among the 3 groups (*P* > .05). There was no significant difference in ODI and VAS before the first operation, length of medical history before the first operation, operation level in the first operation, and interval time between the 2 operations in the LBP group and non-LBP group (*P *> .05). There were significant differences in ODI and VAS before the second operation in the LBP group and the non-LBP group (*P* < .05). There was no significant difference in ODI and VAS before the first and second operation in the LBP group (*P* > .05), and ODI and VAS before the second operation in the non-LBP group were significantly improved compared with those before the first operation, and the difference was statistically significant (*P* < .05). All details were shown in Table 1.

**Table 1:** Comparison of general data in groups

	Low back pain group	Non-low back pain group	Control group	*χ^2^*/*F*/*t*/*Z*	*P*
Gender					
M	7	6	5	2.961	0.228
F	8	4	13		
Age (year)	38～66 (52.20 ± 9.70)	39～65 (52.60 ± 8.66)	34～65 (46.11 ± 8.32)	2.585	0.088
BMI (kg/m^2^)	16～29 (22.27 ± 4.23)	17～29 (22.40 ± 3.95)	15～30 (21.27 ± 4.69)	0.366	0.696
Chronic low back pain before the first operation					
Yes	11	7	/	0.033	0.856
No	4	3			
ODI before the first operation (%)	12～49 (29.67 ± 10.43)	10～42 (31.00 ± 10.88)	/	−0.308	0.761
VAS before the first operation	2～8 (5.47 ± 1.80)	2～8 (5.81 ± 1.87)	/	−0.445	0.660
Length of medical history before the first operation (month)	6～48 (24.87 ± 10.88)	12～60 (31.80 ± 15.60)	/	−1.313	0.202
Level of operation					
L_3/4_	5	3	/	0.031	0.861
L_4/5_	10	7			
ODI before the second operation (%)	20～39 (29.80 ± 6.66)	6～16 (10.00 ± 3.37)	/	8.654	<0.01
VAS before the second operation	4～8 (5.87 ± 1.41)	1.00 (0.75～1.25)	/	17.773	<0.01
Interval between 2 operations (month)	16～45 (27.87 ± 9.40)	18～40 (27.40 ± 6.75)	/	0.135	0.894

### 3.2. Comparison of multifidus histology and TGF-β1 expression between the groups

The CSA of multifidus, muscle fiber CSA/collagen fiber CSA, TGF-β1 immunohistochemical IOD, TGF-β1 immunofluorescence IOD, and gray value ratio (TGF-β1 protein gray value/GAPDH protein gray value detected by Western blot) in the LBP group, the non-LBP group and the control group were in accordance with the normal distribution. There were significant differences in CSA of multifidus, muscle fiber CSA/collagen fiber SA, TGF-β1 immunohistochemical IOD, TGF-β1 immunofluorescence IOD, and gray value ratio among the LBP group, non-LBP group and control group (*P* < .05). There were no significant differences in CSA of multifidus, muscle fiber CSA/collagen fiber CSA, TGF-β1 immunohistochemical IOD, TGF-β1 immunofluorescence IOD and gray ratio between the non-LBP group and the control group (*P* > .05). The CSA of multifidus, muscle fiber CSA/collagen fiber CSA in the non-LBP group and the control group were significantly higher than those in the LBP group (*P *< .05). The TGF-β1 immunohistochemistry IOD, TGF-β1 immunofluorescence IOD, and gray value ratio in the non-LBP group and the control group were lower than those in the LBP group (*P* < .05) (Table 2).

**Table 2:** Comparison of multifidus muscle histology and TGF-β1 expression in the LBP group, the non-LBP group and the control group (minimum to maximum *x ± s*)

	Low back pain group	Non-low back pain group	Control group	*F*	*p*
CSA	799～3670 (2041.80 ± 839.40)	1569～3697 (2962.80 ± 734.51)	1300～3970 (2987.67 ± 762.68)	6.993	<0.01
Muscle fiber CSA/collagen fiber CSA	0.99～4.52 (2.50 ± 1.22)	2.58～6.36 (4.31 ± 1.18)	1.36～8.34 (4.58 ± 2.07)	7.344	<0.01
TGF-β1 immunohistochemistry IOD	789～6470 (3021.20 ± 1666.55)	1056～3098 (1996.20 ± 762.22)	120～3590 (1644.00 ± 953.35)	5.404	<0.01
TGF-β1 immunofluorescence IOD	1236～4950 (3321.47 ± 1210.80)	758～4236 (2204.60 ± 1176.32)	269～3895 (2089.28 ± 1055.60)	5.370	<0.01
Gray value ratio	1.97～6.27 (3.60 ± 1.25)	0.77～3.25 (1.66 ± 0.79)	0.18～2.69 (1.10 ± 0.79)	28.100	<0.01

### 3.3.

**Figure 1.**
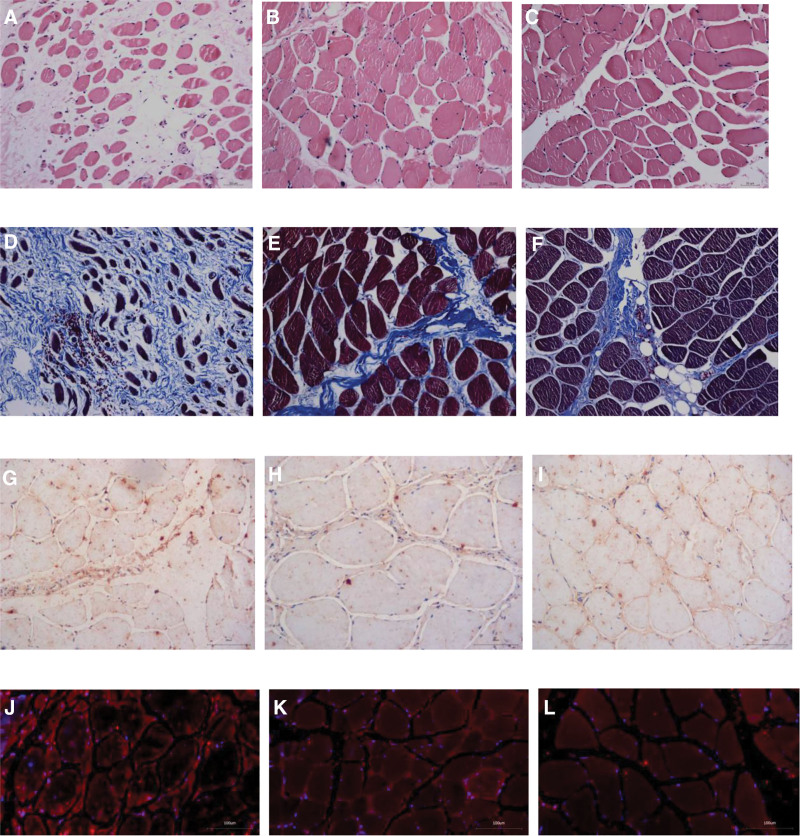
HE staining, Masson’s trichrome staining, TGF-β1 immunohistochemistry, and TGF-β1 immunofluorescence of multifidus in the low back pain group, the non-low back pain group and the control group. A–C: The results of HE staining showed that the CSA of myofiber cells in the multifidus of the low back pain group (A) was significantly reduced, the intercellular distance was widened, and the number of myofiber cells was reduced, and the atrophy of myofiber cells was not obvious in the non-low back pain group (B) and the control group (C), the cell arrangement was still neat, and the cells were compact; D-F: Masson’s trichrome staining showed that in the multifidus of the low back pain group (D), the intercellular distance of dark red muscle fibers was widened, and full with stained blue collagen fiber cells, and the CSA of the latter had exceeded that of the former, showing significant fibrotic changes, the myofiber cells in the non-low back pain group (E) and the control group (F) were arranged neatly, and a small amount of collagen cells infiltrated between myocytes; G-I: TGF-β1 immunohistochemical showed that the brown staining indicated positive TGF-β1. In the low back pain group (G), the brown staining area in the multifidus showed patchy distribution, with positive expression between cells, cell membrane and cells. In the non-low back pain group (H) and the control group (I), the brown area in the multifidus showed punctate distribution, mainly distributed between myocytes; J-L: TGF-β1 immunofluorescence showed that the positive results were red fluorescence, which was similar to the immunohistochemical results, and the positive expression was significantly enhanced in the low back pain group (J), with intracellular and extracellular distribution, and the positive expression was scattered in the non-low back pain group (K) and the control group (L), mainly located between myocytes.

**Figure 2:**
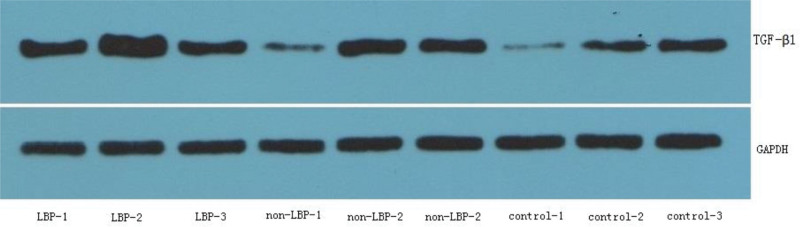
. The expression of TGF-β1 and GAPDH protein in the multifidus by western blot in the 3 groups.

The typical pictures of HE staining, Masson’s trichrome staining, TGF-β1 immunohistochemistry, and TGF-β1 immunofluorescence in the multifidus of the LBP group, the non-LBP group and the control group were shown in Figure 1. The gray value of TGF-β1 protein/GAPDH protein detected by Western blot in multifidus of the LBP group, the non-LBP group and the control group was shown in Figure 2.

### 3.4. Relationship between multifidus morphology, TGF-β1 protein expression and chronic LBP, dysfunction in the LBP group

**Figure 3.**
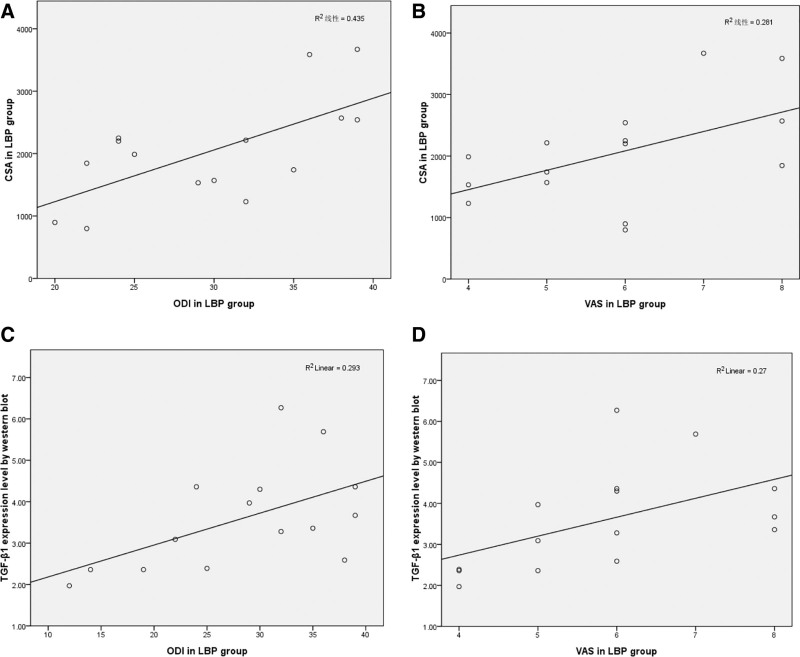
Relationship between multifidus morphology, TGF-β1 protein expression and chronic low back pain, dysfunction in the low back pain group. A. There was a strong correlation between CSA and ODI (*R* = 0.659, *P* < .01); B. There was a strong correlation between CSA and VAS (*R* = 0.530, *P* < .05); C. There was a strong correlation between the TGF-β1 protein and ODI (*R* = 0.542, *P* = .037); D. There was a strong correlation between the expression of TGF-β1 protein and VAS (*R* = 0.520, *P* = .047).

Correlation analysis of the relationship between multifidus morphology, TGF-β1 protein expression and chronic LBP, dysfunction in the LBP group was performed using Pearson correlation coefficient and the results showed a strong correlation between multifidus CSA and ODI (*R* = 0.659, *P* < .01) and VAS (*R* = 0.530, *P *< .05). There was a strong correlation between the expression of TGF-β1 protein and ODI (*R* = 0.542, *P *< .05) and VAS (*R* = 0.520, *P* < .05) in multifidus (Fig. 3).

## 4. Discussion

### 4.1. Epidemiology

A systematic literature review reported that 5% to 36% had recurrence of LBP or leg pain 2 years after laminectomy in patients under 70 years of age with lumbar disc herniation.^[7]^ Skolasky et al^[8]^ performed a prospective study in 260 patients with degenerative lumbar stenosis who underwent lumbar laminectomy with or without fusion, and 29.2% had no change or worsening of pain after 1 year. Another study analyzed 221 patients who received surgery for lumbar disc herniation, and the incidence of persistent LBP after posterior lumbar surgery was 7.2%.^[9]^

### 4.2. Effect of multifidus atrophy on prognosis after posterior lumbar surgery

Skeletal muscle atrophy is associated with increased morbidity and mortality of various diseases and reduced quality of life. Atrophic skeletal muscle is characterized by a reduction or loss of muscle mass, which is accompanied by a reduction in the CSA of muscle fibers, muscle volume, and number of muscle proteins^.[10]^ Studies on the relationship between skeletal muscle atrophy and dysfunction have shown that atrophy reduces the production of contractility by reducing the size of muscle fibers, and changes in fiber type also affects the ability of muscles to continuously contract.^[11]^

In the early stage, scholars conducted comparative studies through paravertebral electrophysiology. As described by Macintosh, there was insufficient evidence to demonstrate a relationship between denervated paraspinal muscle atrophy and postoperative lumbar syndrome in patients undergoing posterior lumbar surgery.^[12]^ In contrast, there are many reasons for unsatisfactory clinical effect after posterior lumbar internal fixation and fusion, of which, paravertebral muscle dysfunction due to atrophy is one of the most discussed. Sihvonen et al^[13]^ reported that the degree of paraspinal muscle denervation was significantly greater in patients with failed back surgery syndrome than in patients with satisfactory surgical results. Rantanen et al^[14]^ also considered that denervated multifidus might be associated with unsatisfactory clinical outcomes. Wilbourn demonstrated that denervated atrophy of paraspinal muscles resulted in significant alterations in spinal biomechanics that might be the cause of LBP.^[15]^

Weber et al^[16]^ analyzed histology and concluded that posterior lumbar surgery caused muscle changes that were not associated with pain or other clinical symptoms. Hence, when the outcomes after lumbar surgery are unsatisfactory, other causes should be considered rather than muscle injury.

With the development of image detection technology and digital technology, the evaluation methods are gradually increasing. Hartwig^[17]^ applied 3-dimensional image to assess the changes of paraspinal muscles in patients with single-level 360° lumbar fusion, and the volume atrophy of paraspinal muscles increased 1 year after surgery, which was considered to be one of the causes of postoperative LBP. Fan proposed that the degree of atrophy was significantly correlated with postoperative LBP and dysfunction through the analysis of imaging changes after open and minimally invasive surgery.^[18]^ Waschke et al proposed a similar opinion that paraspinal muscle atrophy was associated with poor clinical prognosis, which might be a decisive factor in the success of lumbar surgery.^[19]^ In this study, multifidus atrophy occurred in patients with chronic LBP after posterior lumbar surgery, and multifidus CSA was strongly correlated with both ODI and VAS, suggesting that multifidus atrophy was associated with postoperative dysfunction and pain. We believe that multifidus atrophy significantly affects the clinical outcomes after posterior lumbar surgery.

### 4.3. Effect of multifidus fibrosis on the prognosis after posterior lumbar surgery

There are a variety of stem cells in skeletal muscle, of which the most clear significance is muscle satellite cells, which are involved in muscle formation during muscle regeneration. The second is a recently described population of cells called Fibro-adipogenic progenitors (FAPs). FAPs are resident stem cells that have recently been found to play important roles in muscle regeneration, fat infiltration, and fibrosis. These cells are quiescent in healthy muscle and become an important cellular origin for fibrosis and fat infiltration after muscle injury.^[20]^ In multifidus of patients with acute (history <6 months) and chronic (history more than 6 months) LBP, proliferation of FAPs can be observed early, and differentiates into adipocytes and fibroblasts which is the main contributor to fatty infiltration and fibrosis in multifidus muscle. Skeletal muscle fibrosis impairs function. Fibrous tissue limits the migration of cells and hinders the biomechanical properties of normal tissues, and forms an environment not suitable for tissue healing, negatively affecting muscle regeneration, and increasing muscle sensitivity to reinjury, which is considered to be the main cause of muscle weakness and frequent pain.^[21]^

Lehto^[22]^ found that the severity of muscle atrophy was associated with dysfunction at 1-year postoperative follow-up in patients with lumbar disc herniation through a comparative study and proposed that if fibrosis occurred, it could adversely affect the long-term postoperative outcomes.

Posterior lumbar surgery has direct or indirect damage to the multifidus. Muscle has the ability to heal spontaneously after muscle injury, and appropriate fibrosis is necessary for healing, and extracellular components are essential for the integrity of the tissue. However, excessive fibrosis may lead to poor recovery of function. Transforming growth factor is considered to be the main stimulator during fibrogenesis.^[23]^ TGF-β1 is a core molecule regulating skeletal muscle fibrosis, and in some muscular dystrophies, the synthesis and accumulation of extracellular matrix leads to the gradual replacement of functional muscle tissue by connective tissue, with consequent loss of muscle function.

Previous studies showed that TGF-β1 played a key role in inducing muscle fibrosis by increasing profibrotic factors such as collagen and fibronectin.^[24]^ Existing evidence revealed the effects of TGF-β1 on fibrosis and failure of skeletal muscle regeneration and found that the main effects of TGF-β1 overexpression in skeletal muscle were muscle atrophy and endomysial fibrosis.^[25]^ Additionally, the severity of the lesion was related to the total amount of TGF-β1 and the expression of endogenous TGF-β1.

Shahidi et al^[26]^ found that fibrosis gene expression was increased in patients with chronic LBP compared with patients with acute LBP by measuring the expressions of 42 genes associated with myogenesis, atrophy, lipogenesis, metabolism, inflammation, and fibrosis, but there was no significant difference in atrophy, myogenesis, lipogenesis, or inflammatory pathways, which indicated that fibrotic lesion was a key factor leading to chronic LBP. For improving the muscle function, the prevention or reversal of the occurrence of excessive fibrosis should be considered.

No previous studies have analyzed the expression of TGF-β1 in paravertebral muscles after posterior lumbar surgery and its significance. In this study, the expression of TGF-β1 in patients with LBP was higher than that in patients in the non-LBP and the control group. Combined with the morphological observation of Masson staining, the results suggested that multifidus fibrosis was more obvious in patients with chronic LBP. Pearson correlation analysis showed a positive correlation between ODI, VAS and TGF-β1 expression, suggesting that more severe multifidus fibrosis was accompanied by more severe pain and dysfunction.

In summary, patients with chronic LBP after posterior lumbar surgery have atrophy and fibrotic lesions in the multifidus, and the degree of multifidus lesions is closely related to dysfunction and pain, which may be one of the causes of chronic LBP after posterior lumbar surgery.

### 4.4. Limitation

This study has some limitations. First, the sample size in this study is small and may not accurately reflect the overall situation. Large sample size still needs to further confirm the conclusion of this study. Second, although TGF-β1 is one of the factors associated with fibrosis, there are many other factors to consider, and the selection of only a single factor may cause some limitations.

## Author contributions

**Conceptualization:** Dan Pan, Tiansheng Sun.

**Data curation:** Dan Pan, Tiansheng Sun.

**Software:** Dan Pan, Ke Liu.

**Writing—original draft:** Dan Pan.

**Formal analysis:** Ke Liu, Zhicheng Zhang.

**Funding acquisition:** Ke Liu, Zhicheng Zhang.

**Methodology:** Meiyuan Huang, Tiansheng Sun.

**Project administration:** Meiyuan Huang, Zhicheng Zhang.

**Supervision:** Tiansheng Sun.

**Visualization:** Tiansheng Sun.

**Writing—review and editing:** Tiansheng Sun.

**Investigation:** Zhicheng Zhang.

**Resources:** Zhicheng Zhang.

**Validation:** Zhicheng Zhang.
